# Systemic antitumor immune response of doped yttria nanoscintillators under low-dose x-ray irradiation

**DOI:** 10.1126/sciadv.adr4008

**Published:** 2025-03-26

**Authors:** Onur Sahin, Yuri Mackeyev, Geraldine V. Vijay, Soumyabrata Roy, Ashokkumar Meiyazhagan, Yasmin Zahra, Okan Tezcan, Valeria Gonzalez, Belal Abousaida, Holden R. Wagner, Pearl Fernandes, Riaz Mowzoon-Mogharrabi, Bhanu P. Venkatesulu, Cheng-En Hsieh, Joseph B. K. Kim, Subhiksha Raghuram, Xiang Zhang, Kristen A. Miller, Guanhui Gao, Pankaj K. Singh, Sang Hyun Cho, Rao V. L. Papineni, Pulickel M. Ajayan, Sunil Krishnan

**Affiliations:** ^1^ Department of Materials Science and Nanoengineering, Rice University, Houston, TX 77005, USA.; ^2^ Department of Neurosurgery, McGovern Medical School at UTHealth Houston, Houston, TX 77030, USA.; ^3^ Department of Radiation Oncology, Mayo Clinic Florida, Jacksonville, FL 32224 USA.; ^4^ Department of Radiation Oncology, University of Kansas Medical Center, Kansas City, KS 66160, USA.; ^5^ Department of Chemistry, University of California, Berkley, Berkley, CA 94720, USA.; ^6^ Department of Radiation Oncology, University of Pittsburgh Medical Center, Pittsburgh, PA 15219, USA.; ^7^ Division of Radiation Oncology, University of Texas MD Anderson Cancer Center, Houston, TX 77030, USA.; ^8^ PACT & Health LLC, Branford, CT 06405, USA.

## Abstract

Inadequate light penetration in tissues restricts photodynamic therapy to treating only superficial tumors. To enable x-ray–excited photodynamic therapy (XPDT) that targets deep-seated tumors, we synthesized a nanoscintillator-photosensitizer complex containing 5% Eu-doped Y_2_O_3_ fluorescing at 611 nanometers and decorated with SiO_2_ containing the scintillation-coupled photosensitizer methylene blue and a polyethylene glycol coating [PEGylated Y_2_O_3_:Eu@SiO_2_-methylene blue (pYSM)]. When irradiated, pYSMs generate singlet oxygen species in vitro, causing cytotoxicity with hallmarks of immunogenic cell death (calreticulin translocation to the cell membrane). Intravenously administered pYSMs home passively to pancreatic tumor xenografts and, upon 10 gray irradiation, cause significant tumor regression (*P* < 0.01). On combining XPDT with anti-PD1 immunotherapy, a distant nonirradiated tumor also regresses via an increase in intratumoral activated CD8^+^ cytotoxic T cells. Collectively, we advance a systemically delivered XPDT strategy that mediates an antitumor effect in both irradiated and nonirradiated (abscopal) tumors when coupled with immunotherapy, converting an immunologically “cold” tumor to an immunologically “hot” tumor.

## Teaser

X-ray–excited photodynamic therapy with immunotherapy stimulates a local antitumor response and a systemic anticancer effect.

## INTRODUCTION

**Fig. 1.**
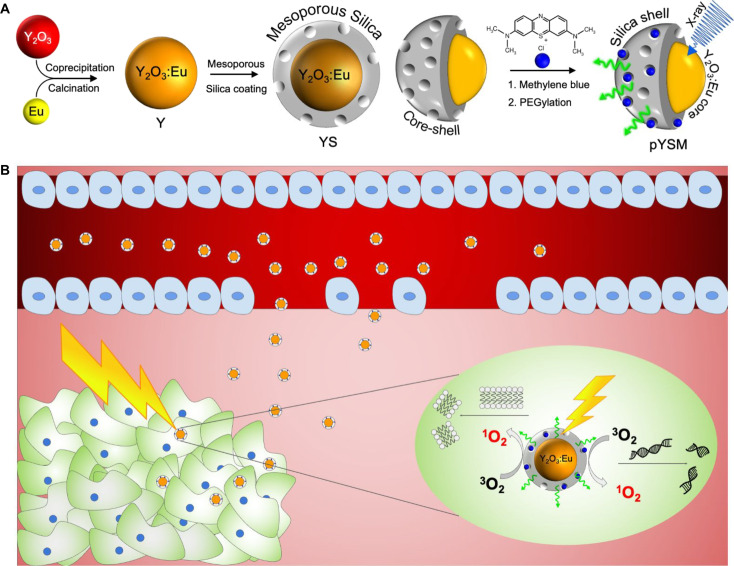
Nanoparticle preparation and XPDT mechanism. (**A**) Schematic preparation of pYSM nanoparticles. (**B**) Illustration of pYSM extravasating through the leaky vasculature to localize in the tumor microenvironment for XPDT. The inset shows pYSM inducing oxidative stress in tumor cells via membrane damage or DNA breakage under x-ray excitation.

Photoacoustic imaging (*1*), photothermal therapy (*2*, *3*), and photodynamic therapy (*4*) are increasingly deployed for the diagnosis and treatment of superficial or endoscopically accessible tissues (*5*). However, the inability of light to traverse beyond 1 cm of tissue thickness remains a major challenge for the clinical deployment of these modalities in the diagnosis and treatment of tumors in deep-seated tissues (*6*). X-ray–excited photodynamic therapy (XPDT) is a recently proposed modality for treating deep tissue tumors (*7*). In XPDT, a scintillating nanoparticle is conjugated to a photosensitizing compound, and under x-ray excitation, the nanoscintillator emits light, which is then absorbed by the photosensitizer to generate reactive oxygen species (ROS) (*8*). These ROS can then induce cell death via oxidative stress through DNA or lipid membrane damage (Fig. 1). Because of the penetration depth and spatial localization of ionizing radiation systems, XPDT has the potential to supplement radiotherapy (RT) and decrease the radiation dose given to patients while improving the cytotoxic efficacy of RT (*9*). A critical factor in the performance of XPDT is the efficiency in ROS generation per unit dose (*10*). Thus, scintillators with a high light yield and photosensitizers with a high singlet oxygen quantum yield are required to produce ROS for a minimal given radiation dose.

In this study, we synthesized a novel XPDT agent, PEGylated Y_2_O_3_:Eu@SiO_2_-methylene blue (pYSM), and demonstrated that XPDT that was performed with pYSM in combination with immune checkpoint blockade promoted a systemic antitumor immune response against solid tumors (Fig. 1, A and B). We chose Y_2_O_3_ doped with europium as a prototype phosphor because of its high light yield under x-ray excitation (*11*), relative ease of nanoscale fabrication, remarkable chemical inertness, and the accepted clinical use of yttria-based compounds (*12*). The photosensitizer was loaded into a mesoporous silica shell to couple the scintillator emission and photosensitizer absorption, this shell was then coated with polyethylene glycol (PEG) chains to increase the circulation time and biodistribution of the agent in the tumor microenvironment (*13*). In addition, whereas prior reports of XPDT agents have used long exposure times with low-energy x-rays [50 kilovolt peaks (kVp)], which do not penetrate well into deep tissues, our results use intravenously administered nanoparticles that accumulate in the tumor and are triggered by clinically relevant, deep-penetrating orthovoltage x-rays (320 kVp) (fig. S1) and define a heretofore unexplored synergy with immunotherapy. An abscopal effect was observed in mice with bilateral tumors where right-sided tumors treated with combination XPDT and immunotherapy also resulted in a significant size reduction of untreated left-sided tumors. To our knowledge, this is the first work assessing the systemic immune response of XPDT combined with immunotherapy. The results found in this study suggest that XPDT in combination with checkpoint blockade could change the immune profile of solid tumors and increase their sensitivity to a systemic antitumor immune response.

## RESULTS

### XPDT agent synthesis

**Fig. 2.**
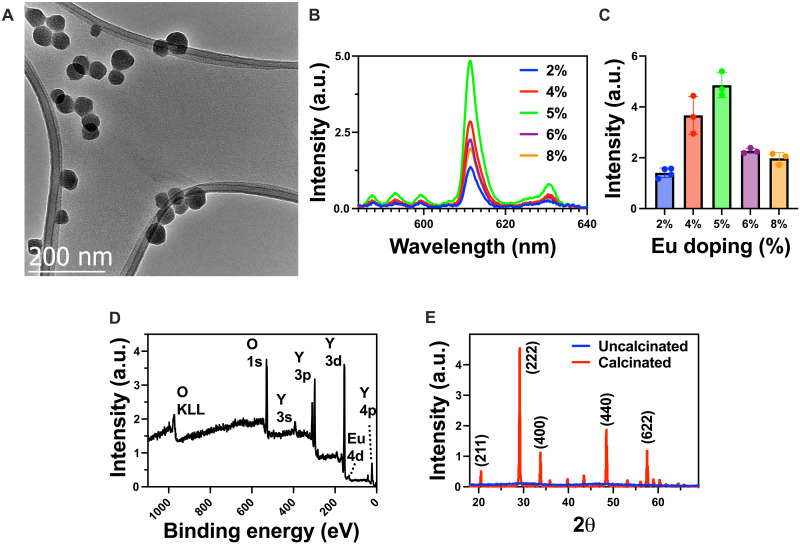
Characterization of Y_2_O_3_:Eu nanoscintillators. (**A**) TEM image of Y_2_O_3_:Eu nanoparticles (scale bar, 200 nm). (**B**) Emission spectra of Y_2_O_3_:Eu nanoparticles from doping concentrations of 2 to 8%. a.u., arbitrary units. (**C**) Relative intensity of Y_2_O_3_:Eu nanoparticles at 611 nm at different doping concentrations. (**D**) XPS survey scan of calcined Y_2_O_3_:Eu nanoparticles. (**E**) XRD patterns of calcined Y_2_O_3_:Eu nanoparticles.

Scintillating Y_2_O_3_:Eu nanoparticles were synthesized via an aqueous coprecipitation method. YCl_3_ and EuCl_3_ were mixed in deionized (DI) water with urea as a precipitating agent and 50-kDa polyvinyl pyrrolidone as a capping agent to control the size and polydispersity of the nanoparticles (*14*, *15*). The resulting rare earth hydroxycarbonates were calcined in open air to yield the scintillating binary oxide. A custom-built optical setup was used to measure the light output of the nanoscintillators excited by 320-kVp x-rays (figs. S2 and S3). To optimize the light yield of the scintillators, the europium doping concentration was optimized. As seen in Fig. 2A, transmission electron microscopy (TEM) images show that the resulting binary oxide nanoparticles are well segregated and have a uniform diameter of ~50 nm. High-resolution TEM also showed a characteristic plane along the [101] zone axis because of the typical orientation of planes (200) and (220) with *d*-spacings of 0.51 and 0.8 nm, respectively (fig. S4). Figure 2B shows the radioluminescence spectra of the Y_2_O_3_:Eu nanoparticles under x-ray excitation. The peak near 611 nm is due to the ^5^D_0_ → ^7^F_2_ transition, which is characteristic of the europium dopant and remains consistent across different doping concentrations (*11*). Figure 2C shows the relationship between the doping concentration of europium and the resulting light yield from 2 to 8% europium doping. When the doping concentration is low, adding more dopant ions increases the light yield. Beyond 5% doping, the light yield begins to decrease as dopant ions are added, which can be due to a self-quenching process where the europium luminescence centers absorb each others’ light emission, resulting in an overall decrease in light yield (*16*). X-ray photoelectron spectroscopy (XPS) and x-ray diffraction (XRD) were performed (Fig. 2, D and E) to further confirm the presence of europium and the crystallinity of the yttria lattice after calcination (fig. S5) (*17*). XRD peaks matched cubic phase Y_2_O_3_ (JCPDS #71-0099).

**Fig. 3.**
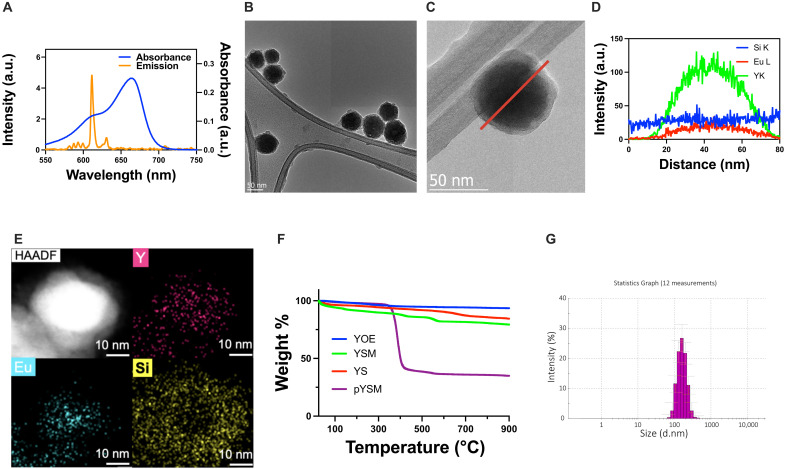
Characterization of the pYSM XPDT agent. (**A**) Ultraviolet-visible absorption of a methylene blue photosensitizer and overlap with Y_2_O_3_:Eu emission. (**B** and **C**) TEM image of the pYSM core-shell complex (scale bar, 50 nm). (**D**) EDS line scan across the pYSM particle. (**E**) EDS color mapping of the XPDT agent with a high-angle annular dark-field image (yttrium in pink, europium in teal, and silicon in yellow; scale bar, 5 nm). (**F**) TGA of Y_2_O_3_:Eu, Y_2_O_3_:Eu@SiO_2_ (YS), Y_2_O_3_:Eu@SiO_2_ with methylene blue (YSM), and PEGylated Y_2_O_3_:Eu@SiO_2_ with methylene blue (pYSM). (**G**) DLS of PEGylated pYSM particles run in triplicate.

XPDT requires a photosensitizer that can absorb the light emitted from a scintillator to generate ROS. In this work, methylene blue was chosen as the photosensitizer because of the substantial overlap between the absorption band of methylene blue and the emission maxima of Y_2_O_3_:Eu (Fig. 3A). To form a photosensitizer-scintillator complex, Y_2_O_3_:Eu was coated with a mesoporous silica shell using a modified version of the Ströber process with methylene blue present in the reaction mixture (*18*). TEM images revealed the formation of a silica shell and a final average particle diameter of ~80 nm (Fig. 3B). In addition, energy dispersive x-ray spectroscopy (EDS) line scan performed across the particle showed the expected core-shell particle morphology with the prominence of yttrium compared to europium within the center of the particle and the relatively constant signal of silicon (Fig. 3C). Similarly, EDS color mapping revealed the uniform distribution of europium and yttrium within the core of the particle (Fig. 3D) with a uniform SiO_2_ coating. Thermogravimetric analysis (TGA) of the XPDT agent at different synthetic stages showed that the methylene blue loaded to the nanoparticle contributes about 10% of the weight of YSM as well as the successful PEGylation of pYSM (Fig. 3E), which was added to increase the circulation time of the XPDT construct in the vasculature. Dynamic light scattering (DLS) analysis of pYSM showed hydrodynamic diameters of 176.3 ± 35 nm in water, 162.4 ± 37 nm in phosphate-buffered saline (Fig. 3F), and 162.5 ± 52.6 nm in 2% bovine serum albumin. These remained relatively unchanged at 24 and 72 hours (fig. S6). The corresponding zeta potentials were −3.2 ± 4.6, −3.09 ± 3.8, and −3.6 ± 5.2 mV in water, phosphate-buffered saline, and bovine serum albumin, respectively. These also remained stable in solution at 24 and 72 hours (fig. S7). Both these metrics suggest that pYSMs are stable and could be used under physiological conditions over a prolonged duration. The narrow size distribution ensures excellent monodispersity and extravasation through the leaky tumor vasculature, allowing for uptake and retention in the tumor by the enhanced permeability and retention (EPR) effect (*19*).

### In vitro XPDT studies

**Fig. 4.**
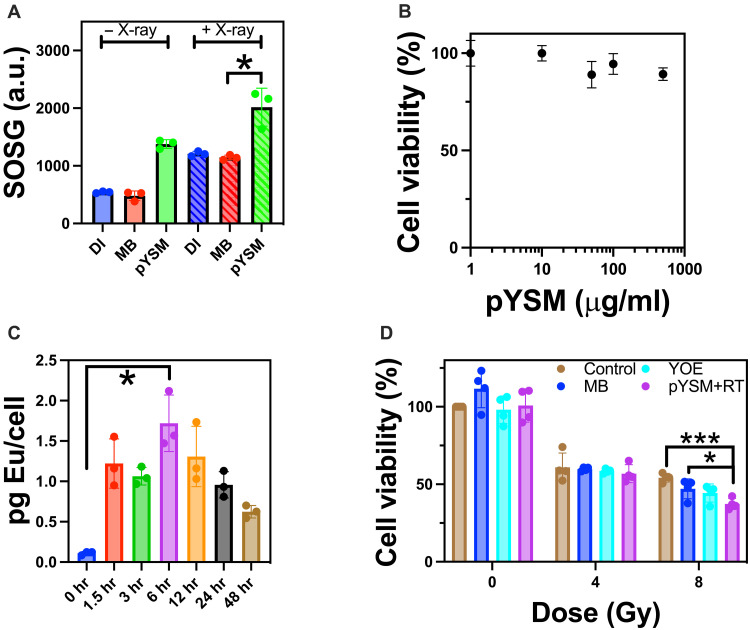
In vitro characterization of XPDT. (**A**) SOSG assay of pYSM, methylene blue only (MB), and background DI water (DI) with and without irradiation. (**B**) MTS assay showing the cellular viability of pYSM. (**C**) Cellular uptake of the pYSM agent performed in triplicate at varying time points. hr, hours. (**D**) In vitro comparison of XPDT versus controls: RT only (control), methylene blue + RT, Y_2_O_3_:Eu (YOE) + RT (*P < 0.05 and ***P < 0.001).

To characterize x-ray–excited ROS generation, a singlet oxygen sensor green (SOSG) assay was performed with pYSM compared to methylene blue and DI water as controls (*20*). As seen in Fig. 4A, the pYSM group produced the largest statistically significant amount of singlet oxygen upon irradiation compared to controls, showing that Y_2_O_3_:Eu is required to activate methylene blue upon x-ray excitation. Notably, there was no appreciable increase in temperature ruling out a photothermal mechanism of action (fig. S8). Encouraged by these results, we incubated the pYSM agent at varying concentrations with murine Panc02 pancreatic cancer cells to measure the toxicity of the XPDT agent. As seen in Fig. 4B, cells remained viable at concentrations above 100 μg/ml. In addition, cellular uptake studies were performed via inductively coupled plasma mass spectrometry (ICP-MS), which showed that 6 hours of incubation resulted in the maximal pYSM concentration within cells (Fig. 4C). Analysis of variance followed by Tukey’s post hoc test was performed, showing a statistically significant difference in cellular uptake between 0 and 6 hours of incubation. To test the in vitro consequences of XPDT-mediated singlet oxygen production, we treated Panc02 cells with pYSM particles and respective controls and split them into irradiated and nonirradiated groups (Fig. 4D). While no noticeable cell death occurred in the nonirradiated group, cell viability decreased significantly after a dose of 8–gray (Gy) irradiation in cells cultured with pYSM when compared to the control and pYSM-only groups, suggesting that pYSM could be used as an XPDT agent.

### In vivo XPDT studies

**Fig. 5.**
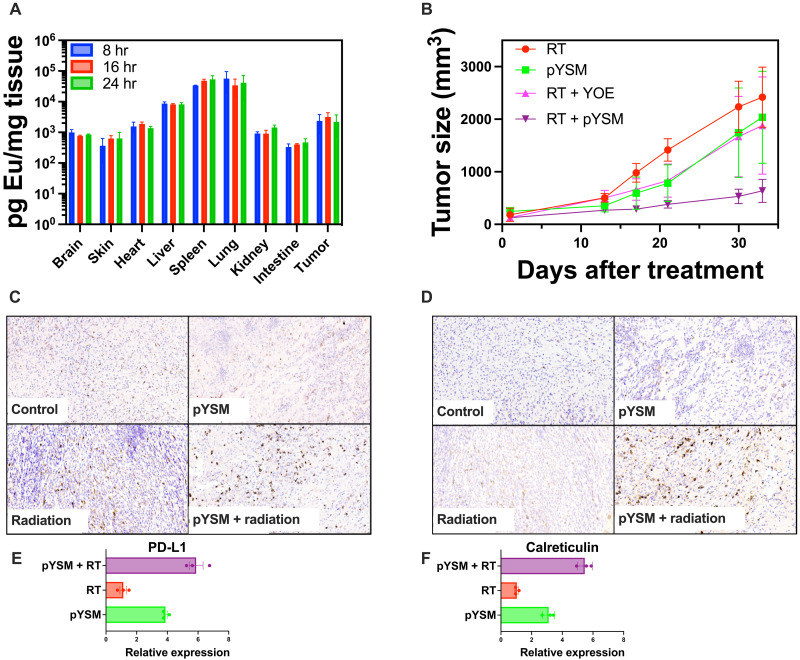
In vivo XPDT experiments. (**A**) Biodistribution assay shows the successful delivery of pYSM to the tumor. (**B**) In vivo XPDT shows significant reduction in tumor size with pYSM + radiation versus radiation alone. (**C**) Representative images of tumor sections stained for CD8 demonstrating increased infiltration of immune cells in the tumor microenvironment. (**D**) Representative images of tumor sections stained for CD8 demonstrating increased infiltration of immune cells in the tumor microenvironment. (**E**) In vitro calreticulin expression increased in cells treated with pYSM + radiation compared to controls. (**F**) In vitro PD-L1 expression increased in cells treated with pYSM + radiation compared to controls.

To be effective as an XPDT agent, systemically delivered pYSM must accumulate in the target tumor. In a biodistribution analysis conducted in mice with subcutaneous Panc02 tumors, normal organs and tumors were harvested at 8, 16, and 24 hours following the intravenous administration of pYSM. Tumor uptake was noted to be relatively constant at all time points with no statistically significant difference in uptake, while the liver, spleen, and lungs showed high uptake as well (Fig. 5A). On the basis of this, we proceeded to perform a tumor regrowth delay experiment with pYSM administered intravenously 24 hours before irradiation with a single dose of 10 Gy. A statistically significant decrease in tumor volume was noted in the pYSM + radiation group compared to radiation alone (Fig. 5B). Immunohistochemical analysis of the tumors harvested from these mice showed an increase in the CD8^+^ and CD4^+^ T cells in the XPDT-treated tumors (Fig. 5, C and D). Surprised by this finding, we went back to test the in vitro immunogenicity of XPDT and observed that XPDT increased the expression of both calreticulin and programmed death ligand 1 (PD-L1) on the surface of Panc02 cells when compared to just RT or only particle incubation. This indicated that not only was there an immunogenic cell death response (*21*), but there was also an immunosuppressive (*22*), yet druggable, phenotype activated as well (Fig. 5, E and F). This experiment suggested that the antitumor activity of XPDT therapy could be enhanced if coupled with immunotherapy aimed at boosting the immune response mounted against the tumor. Mouse weights were also separately tracked for the control, RT-only, pYSM-only, and XPDT groups and showed no appreciable difference across the treatment groups, suggesting no overt toxicity of the pYSMs (fig. S9).

### XPDT combined with immunotherapy

**Fig. 6.**
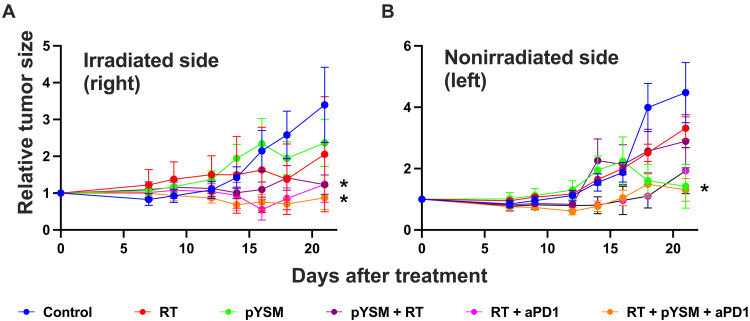
Combination of XPDT with immunotherapy. (**A**) Tumor growth delay curves of the irradiated right-sided tumor. (**B**) Tumor growth delay curves of the nonirradiated left-sided tumor (*P < 0.05 compared to the control group).

To test this hypothesis, ovalbumin-expressing Panc02 (Panc02-Ova) cells were subcutaneously implanted in both thighs of immune-competent C57BL/6 mice and treated with a combination of XPDT and anti-PD1 immunotherapy. The tumors on the right thigh were treated, but the tumors on the left thigh were not. These nonirradiated tumors served as surrogates for distant or metastatic disease, allowing us to interrogate abscopal responses driven by the activation of a robust systemic immune response. Our analysis of the right-sided tumor showed that pYSM + RT significantly decreased the tumor growth, as previously demonstrated (Fig. 5B), but also that this tumor growth inhibition was significantly augmented by the addition of anti-PD1 therapy (Fig. 6A). Compared to the control group, two groups of mice had statistically significant regression in tumor size: mice given either XPDT or XPDT + anti-PD1 therapy. Similarly, the left-sided tumors in the XPDT + anti-PD1 group had a statistically significant decrease in tumor volume, suggesting that a robust systemic immune response was activated (Fig. 6B).

**Fig. 7.**
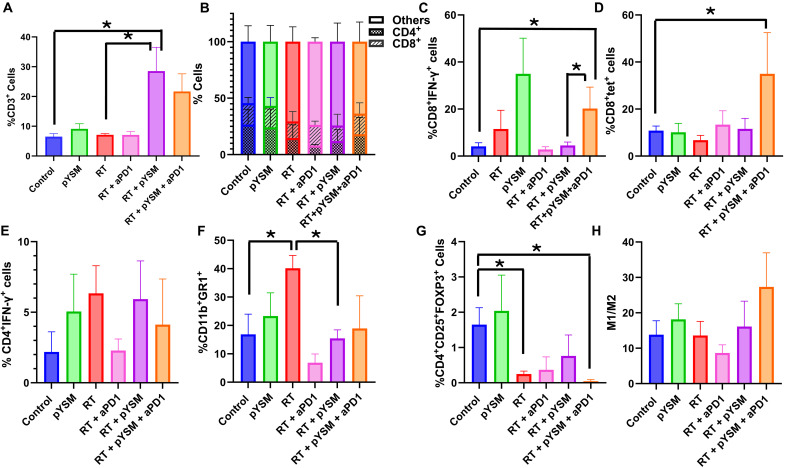
Radiation alone and in combination with YSM and anti-PD1 therapy alters the tumor immune microenvironment of the irradiated right tumor. The right tumors were immunophenotyped, and the changes in (**A**) CD3^+^ T cells, (**B**) CD4^+^ and CD8^+^ T cells, (**C**) activated CD8^+^ T cells, (**D**) antigen-specific CD8^+^ T cells, (**E**) T_H_1 cells, (**F**) MDSCs, (**G**) T_regs_, and (**H**) M1/M2 macrophage ratio were quantified and graphed (*P < 0.05).

To further characterize this effect, we performed immunophenotyping of tumor-infiltrating lymphocytes harvested from both tumors using flow cytometry. In the treated right-sided tumors, XPDT therapy significantly increased T cell infiltration in the tumor, as indicated by the significant increase in CD3^+^ T cells (inclusive of CD4^+^ and CD8^+^ T cells) in the RT + pYSM–treated tumors when compared to the control group (Fig. 7, A and B). While the addition of anti-PD1 therapy to RT + pYSM did not significantly increase the infiltration of tumors by T cells, it increased the proportion of the activated CD8^+^IFN-γ^+^ T cells and elicited a trend toward increased antigen-specific CD8^+^tet^+^ T cells (Fig. 7, C and D). Analysis of the immunosuppressive cell populations showed that radiation by itself and RT + pYSM + aPD1 decreased the number of CD4^+^CD25^+^FOXP3^+^ regulatory T cells (T_regs_) when compared to untreated control tumors (Fig. 7G). While there were modest, nonstatistically significant differences in the proportion of interferon-γ (IFN-γ)–secreting CD4^+^ T cells [T helper 1 (T_H_1) cells] between the groups (Fig. 7E), there was a significant decrease in the CD11b^+^GR1^+^ myeloid-derived suppressor T cell (MDSC) population in between the RT-only group versus the control and RT + pYSM groups (Fig. 7F). While anti-PD1 therapy slightly increased the M1/M2 macrophage ratio in treated tumors, no statistically significant response was observed (Fig. 7H).

**Fig. 8.**
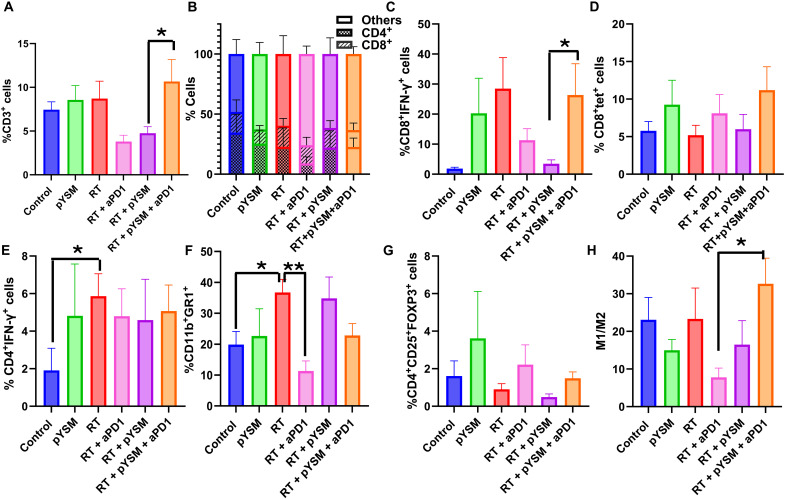
Radiation alone and in combination with YSM and anti-PD1 therapy alters the tumor immune microenvironment of the nonirradiated left tumor. The left tumors were immunophenotyped, and the changes in (**A**) CD3^+^ T cells, (**B**) CD4^+^ and CD8^+^ T cells, (**C**) activated CD8^+^ T cells, (**D**) antigen-specific CD8^+^ T cells, (**E**) T_H_1 cells, (**F**) MDSCs, (**G**) T_regs_, and (**H**) M1/M2 macrophage ratio were quantified and graphed (*P < 0.05).

Analysis of the left tumors, which were not irradiated, showed that neither the RT group nor the RT + pYSM group showed an increase in the CD3^+^ population (Fig. 8A). However, anti-PD1 in combination with radiation and pYSM significantly increased the infiltration of CD3^+^ T cells (inclusive of CD4^+^ and CD8^+^ T cells) in the nonirradiated tumors compared to nonirradiated tumors in the pYSM + RT group (Fig. 8, A and B). Anti-PD1 therapy in combination with RT and pYSM significantly increased the activated CD8^+^IFN-γ^+^ T cells when compared to the RT + pYSM–treated counterparts (Fig. 8C). However, there was no statistically significant increase in antigen-specific T cells in the left tumors of mice between the different treatment groups (Fig. 8D). None of the treatment groups significantly altered the T_reg_ population in the nonirradiated tumors (Fig. 8G). Similar to the irradiated right tumor, RT significantly increased the population of CD11b^+^GR1^+^ MDSCs, but unlike the irradiated tumors, an increase in IFN-γ–secreting T_H_1 cells was also noted in the RT group in the tumor microenvironment (Fig. 8, E and F). When comparing the RT alone and RT + aPD1 groups, the addition of anti-PD1 therapy decreased MDSCs in nonirradiated (left) tumors (Fig. 8F). However, none of the other treatment groups significantly altered the MDSC population in the tumor microenvironment of the nontreated tumor compared to the control (Fig. 8F). Notably, the M1/M2 macrophage ratio in the left tumor was significantly higher in the RT + pYSM + aPD1 group compared to the RT + aPD1 group (Fig. 8G). Collectively, these results suggest a shift in the tumor immune microenvironment favoring a pro-inflammatory antitumor phenotype when XPDT is coupled with immunotherapy.

## DISCUSSION

In summary, we have synthesized a novel XPDT agent, which can efficiently generate ROS upon x-ray irradiation. We showed that pYSM can induce cell death in vitro upon x-ray excitation as well as increase immunogenic cell death and prime cells for immune checkpoint blockade. In vivo, we showed that the combination of XPDT with immunotherapy can not only reduce the tumor burden of solid tumors but can also stimulate an antitumor immune response to mediate abscopal responses. These effects were observed with intravenously administered nanoparticles, at clinically relevant energies, doses, and dose rates of radiation, and with careful characterization of the immune effect. Consequently, this represents a previously unexplored and clinically translatable paradigm for the use of nanophosphors as an in situ x-ray radiation–triggered beacon that can illuminate photosensitizers in deep-seated tumors to generate antitumor effects in both irradiated tumor and nonirradiated tumor. While nontumor tissues take up some nanoparticles, XPDT is only effective when radiation dose from a collimated x-ray beam overlaps with tissue harboring pYSM, thus minimizing off-target toxicity.

In similar studies performed by other groups, scintillating compounds have been coupled to photosensitizers for x-ray–triggered photodynamic effects in vitro, but in vivo translation has relied on intratumoral injections. Nanoscintillators used have included fluorides (*23*–*28*), sulfides (*29*, *30*), and aluminates (*31*, *32*), which are then coupled to photosensitizers for XPDT. However, these works often use 50-kVp mini–x-ray sources, which have much lower energies than those used in clinical RT and have required radiation exposure times over an hour (*33*–*40*). While these studies report low radiation doses that suggest clinical utility, the low x-ray energies do not penetrate well into deep tissue environments when compared to clinical x-rays. In addition, many of these studies perform XPDT after intratumoral injections of their agent, which may limit their clinical feasibility in treating deep tissue tumors. Last, to the best of our knowledge, the immunogenicity of XPDT in promoting a systemic immune response against tumors has not been explored in these previous works. This transformation can be viewed from the vantage point of the categorization of tumors by Chen and Mellman as “immune inflamed” (or “hot”) and “immune noninflamed” (or “cold”) on the basis of the abundance of activated immune cells and pro-inflammatory cytokines in the former and the exclusion or paucity of activated immune cells, with possibly a relative abundance of regulatory or suppressive cell types, and more anti-inflammatory cytokines in the latter (*41*). Seen in this light, the ability of XPDT to convert immune-cold tumors to immune-hot ones is especially beneficial in tumors that have shown little clinical response to immune checkpoint blockade therapy and often present with occult distant metastases such as pancreatic cancer.

Our studies used pYSMs that were not designed for active targeting to tumor cells via surface decoration with peptides, antibodies, or other biomolecules that bind to tumor-specific cell surface targets. Therefore, their selectivity for the tumor derives from the EPR effect of tumors and the collimation of the x-ray beam to just the tumor while sparing adjacent normal tissues. Admittedly, the size of nanoparticles plays a key role in EPR-mediated accumulation in tumors with interendothelial gaps as wide as 380 to 780 nm in a study (*42*) and 400 to 600 nm in another (*43*). Size regimes similar to nab-paclitaxel (Abraxane), a 130-nm albumin-bound formulation of paclitaxel particles used routinely in the treatment of pancreatic cancer, should accumulate passively via the EPR effect (*44*, *45*) and are likely to be more representative of human tumor vasculature than preclinical models, which may not translate as well to human scenarios. Further studies looking at the added value of active targeting of the tumor may be warranted to further minimize off-target toxicity. It is worth noting, however, that in a pooled analysis of over a hundred studies analyzing intravenous nanoparticle delivery to tumors, active targeting strategies only increased the delivered dose to tumors from 0.6% injected dose to 0.9% injected dose (*46*). Additional studies exploring the biocompatibility of pYSMs are needed before contemplating translation to clinical scenarios. The biocompatibility of core-shell constructs where SiO_2_ has been used as an epilayer over a core nanoparticle has been described extensively in the literature (*47*, *48*). Nonetheless, dedicated studies will need to address the long-term safety, toxicity, and biocompatibility of pYSMs in vivo.

By overcoming the limited penetration depth of traditional photodynamic therapy, XPDT expands the applicability of PDT to tumors heretofore considered untreatable. The widespread use of radiation for the treatment of cancers, the relative biocompatibility of components of our construct, and the potential to simulate a robust immune response pave the way for clinical translation. Furthermore, immune activation is especially meaningful for pancreatic cancers and other aggressive cancers, where seemingly localized tumors harbor micrometastases, circulating and disseminated tumor cells, and occult metastatic disease. Together, our approach is a multidimensional attack on tumors that could halt multiple avenues of tumor progression.

## MATERIALS AND METHODS

### Synthesis of pYSM

To synthesize Y_2_O_3_:Eu nanoparticles, DI water (90 ml) was heated to 95°C before adding yttrium chloride hexahydrate (140 mg), europium chloride hexahydrate (8 mg), 50-kDa polyvinyl pyrrolidone (4 g), and urea (16.2 g). The solution was stirred at 1000 rpm for 10 min at 95°C and then centrifuged with DI water before drying in an oven at 70°C overnight. The precursor powder was then calcined at 1000°C in air for 6 hours. YSM core-shell particles were then fabricated by mixing the Y_2_O_3_:Eu powder with tetraethoxysilane (350 μl, Thermo Fisher Scientific), methylene blue [20 ml of 1.4% (w/v) in 95% ethanol, Sigma-Aldrich], and 25% ammonium hydroxide (0.50 ml) in ethanol (80 ml) for 6 hours. The resulting silica particles were washed three times with ethanol via centrifugation, then resuspended in 10 ml of ethanol, and mixed with 5000–molecular weight methoxy PEG trimethylsilane (20 mg, JenKem Technology) for 24 hours.

### Nanoparticle characterizations

XRD measurements were performed on a Rigaku SmartLab X-ray Diffractometer using a Cu anode and a tube voltage and current of 40 kVp and 40 mA, respectively. TEM images were captured using a 200-kV JEOL 2100 TEM field emission gun. Radioluminescence spectra were recorded via a custom setup (fig. S4) using a USB4000 Ocean Optics spectrometer and a 320-kVp x-ray source spaced 35 cm from the powder sample. TGA was performed using a Mettler Toledo TGA/DSC 3+ system. Using a ramp rate of 10°C/min, samples were heated from 25° to 900°C under air. XPS was performed using a PHI Quantera XPS via a 50-W, 15-kV monochromatic Al Kα x-ray source with a 200-μm diameter. EDS color mapping was characterized using a Titan Themis S/TEM. DLS measurements were performed in DI water using a Malvern Zen 3600 Zetasizer. For SOSG characterizations, 3 mM SOSG in ethanol (100 μl) was mixed with YSM (10 μg in 100 μl), Y_2_O_3_:Eu (10 μg in 100 μl), or 1 μM methylene blue in DI water (100 μl). Groups were prepared in triplicate, a 320-kVp x-ray source was used to irradiate samples, and then the 525-nm emission fluorescence of all samples was measured using 504 nm as an excitation source.

### In vitro characterizations

pYSM biocompatibility was measured using the Panc02 murine pancreatic cancer cell line. One thousand Panc02 cells were incubated in 96-well plates for 24 hours before adding either fresh media or nanoparticles at different concentrations. Sixteen wells were cultured for each concentration. A Promega 3-(4,5-dimethylthiazol-2-yl)-5-(3-carboxymethoxyphenyl)-2-(4-sulfophenyl)-2*H*-tetrazolium (MTS) assay was then performed 48 hours later according to the manufacturer’s protocol. XPDT characterizations were also performed using the Panc02 cell line. One thousand Panc02 cells were incubated with nanoparticles as above, media were changed after 24 hours, and cells were irradiated using a 320-kVp x-ray source. Four wells were prepared for each group. MTS assay was then performed 24 hours after irradiation. To measure the in vitro immunogenicity of XPDT, Panc02 cells were cultured in T75 flasks, allowed to reach 80% confluency, and divided into control, pYSM alone, radiation alone, and pYSM + radiation with three flasks prepared for each group. The cells were treated with pYSM for 24 hours, the media were changed, and then cells were irradiated with a single dose of 8 Gy using a 320-kVp x-ray source. Forty-eight hours after cell irradiation, the cells were trypsinized and collected for flow cytometry. The cells were washed twice with phosphate-buffered saline and then aliquoted to 1 × 10^6^ cells per tube. The cells were then stained with primary antibodies diluted in fluorescence-activated cell sorting buffer for 45 min in ice and then anti-mouse primary antibodies directed against calreticulin and PD-L1. Zombie aqua was used as a live/dead discriminator to account for autofluorescence from dead cells. The cells were run through a Gallios 561 flow cytometer, and the collected data were analyzed using Kaluza software.

### In vivo characterizations

All animal experiments were performed in accordance with MD Anderson (protocol MDA-00001163) and Mayo Clinic (protocol Mayo A00004800-19) animal research policies. Panc02-Ova tumors were bilaterally implanted on the right and left thighs of C57BL/6 mice. The tumor-bearing mice were divided into five groups with 12 mice in each group. The mice in the control group did not receive any treatment, whereas the tumors on the right thigh of the mice in the RT-only group were irradiated at 10 Gy. The mice belonging to the pYSM-only, pYSM + RT, and pYSM + RT + anti-PD1 groups were administered 50 mg of pYSM/kg of mouse intravenously via the tail vein. The tumors on the right thigh of the mice belonging to the pYSM + RT and pYSM + RT + anti-PD1 groups were irradiated with 10 Gy of radiation 24 hours following the administration of pYSM. The mice in the pYSM + RT + anti-PD1 group were treated with three doses of InVivoMab anti-mouse PD1 (200 μg; clone: RPM1-14) intraperitoneally every 3 days. The tumors on both sides of the mice were measured on alternate days and graphed. The mice were euthanized at the moribund state. The tumors from the mice euthanized between 10 and 20 days after the last dose of anti-PD1 were harvested and processed for immunophenotyping flow cytometry analysis using the Miltenyi Biotec Mouse Tumor Dissociation kit (catalog no. 130-096-730). The processed tumors were counted and stained for markers (tables S1 and S2) with gating strategies shown in table S3. The flow cytometry data were analyzed using FlowJo and graphed using GraphPad Prism. CD4^+^ and CD8^+^ immunohistochemistry staining was performed by preparing tissue blocks of formalin-fixed and paraffin-embedded tumor specimens. First, the presence of tumor was confirmed using hematoxylin and eosin staining. Slides were hydrated to DI water followed by antigen retrieval (pH 9.0). Slides were blocked with 3% hydrogen peroxide, rinsed with tris-buffered saline, and incubated for 30 min in either CD4 (183685, Abcam) or CD8 (209775, Abcam), followed by another buffer rinse. The slides were incubated for 15 min in anti-rabbit immunoglobulin G (DS9800, Leica), rinsed, counterstained in hematoxylin, dehydrated, and covered in coverslips.

### Inductively coupled plasma analysis of tissue samples

The europium content of in vitro and in vivo samples treated with nanoparticles was quantified by ICP-MS after dissolving samples in concentrated nitric acid. Briefly, the tissue samples were freeze dried for 48 hours, then carefully weighed, and solubilized by treatment with nitric (Thermo Fisher Scientific, A467-1) and hydrochloric (Thermo Fisher Scientific, A466-1) acids under a fume hood. Solubilization of each was performed as follows. Initially, nitric acid (2.5 ml) was added to the sample and allowed to stand for 24 hours at room temperature. This was followed by the addition of hydrochloric acid (0.5 ml), after which the vial was closed by a screw cap with a polytetrafluoroethylene preslit liner and kept on top of a hot plate at 90°C for 48 to 240 hours until the liquid inside became completely clear. The vial was cooled to room temperature, the cap was carefully opened, and the open-top vial was allowed to stand on the hot plate at 120°C until the volume content reduced to ~0.5 ml. Then, the residue was diluted with 2% hydrochloric acid in DI water, and the final volume was adjusted to 10 ml. The solutions were filtered using 0.2-μm glass microfiber syringe filters (Whatman PLC, Florham Park, NJ) into 15-ml PP Eppendorf tubes and subjected to ICP-MS analysis using a quadrupole inductively coupled plasma PerkinElmer NexION 2000 B Mass Spectrometer to estimate the amount of europium. Eu concentrations were calculated from the regression equation built with a set of standards with concentrations of 1 × 10^9^ to 1 × 10^4^ g/liter prepared by diluting the Eu primary standard (998 mg/liter, VWR). A Rb^+^ internal standard (0.10 μg/liter) was added to the blank, all calibration standards, and samples. The Rb^+^ standard was prepared by diluting the Rb^+^ primary standard (1000 mg/liter, Millipore Sigma) in a PP volumetric flask and diluting to the desired volume with DI water.

### Statistical analyses

All statistical analyses were performed using GraphPad Prism software. Student’s *t* test was used for all comparisons except for the in vivo XPDT + immunotherapy experiment where analysis of the variance and Tukey’s range test were used to find statistically significant pairs.
