# Supplementary material for: Systemic antitumor immune response of doped yttria nanoscintillators under low-dose x-ray irradiation
Source: Sci Adv. 2025 Mar 26;11(13):eadr4008. doi: 10.1126/sciadv.adr4008 (PMC11939067; doi:10.1126/sciadv.adr4008)
Supplement: Supplementary file 1 — Figs. S1 to S9 Tables S1 to S3 [file sciadv.adr4008_sm.pdf]

Science Advances
AAAS

Supplementary Materials for

Systemic antitumor immune response of doped yttria nanoscintillators under low-dose x-ray irradiation

Onur Sahin *et al.*

Corresponding author: Sunil Krishnan, sunil.krishnan@uth.tmc.edu

Sci. Adv. **11**, eadr4008 (2025)
DOI: 10.1126/sciadv.adr4008

This PDF file includes:

Figs. S1 to S9
Tables S1 to S3

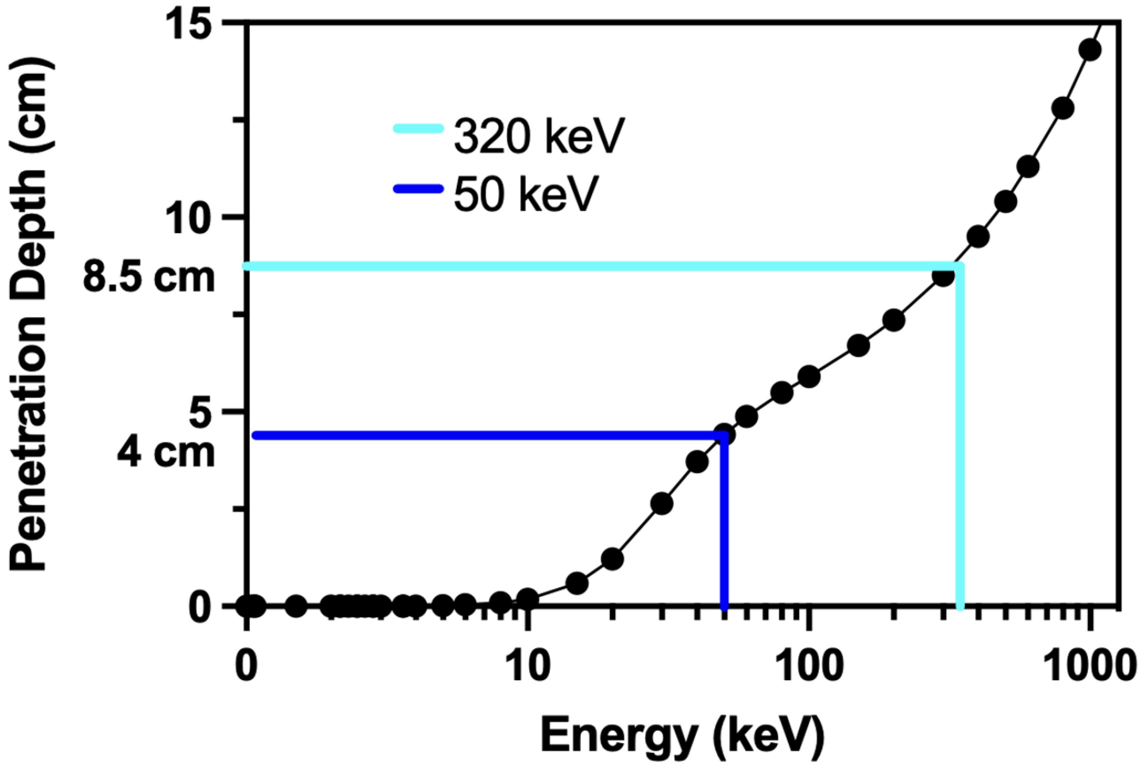

Fig. S1. The penetration depth of X-rays in soft tissue as a function of X-ray energy. Penetration depths were calculated from the inverse of mass attenuation coefficient values of ICRU-44 soft tissue from the NIST database.

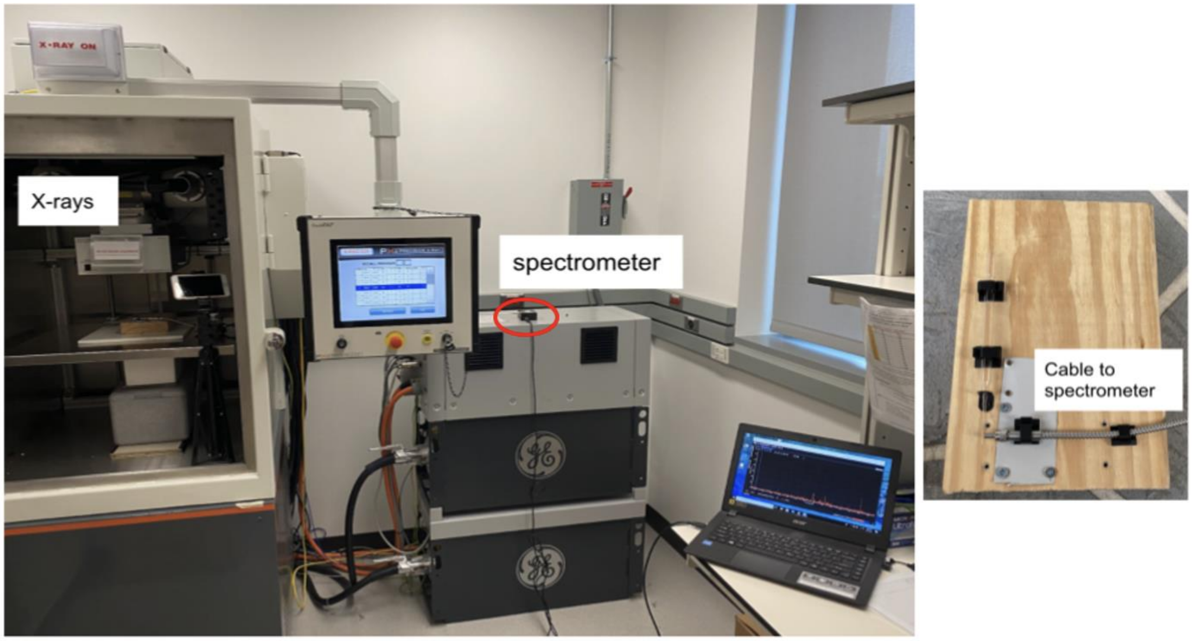

Fig. S2. Custom setup to measure the radioluminescence of Y_2O_3:Eu nanoparticles. Y_2O_3:Eu nanoparticle powder in a cuvette was placed in a holder attached to a fiber optic cable connected to an Ocean Optics USB4000 spectrometer using OceanView software

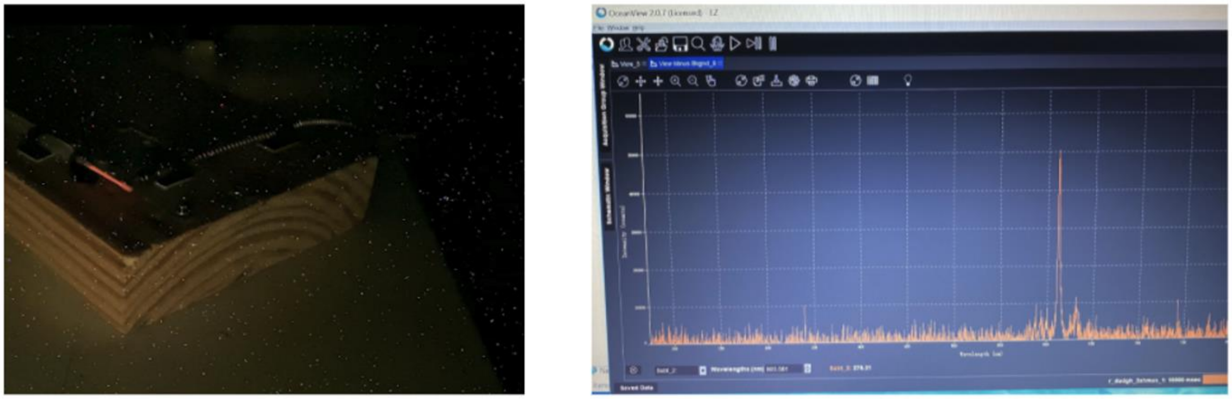

Fig. S3. Image of particles scintillating in setup

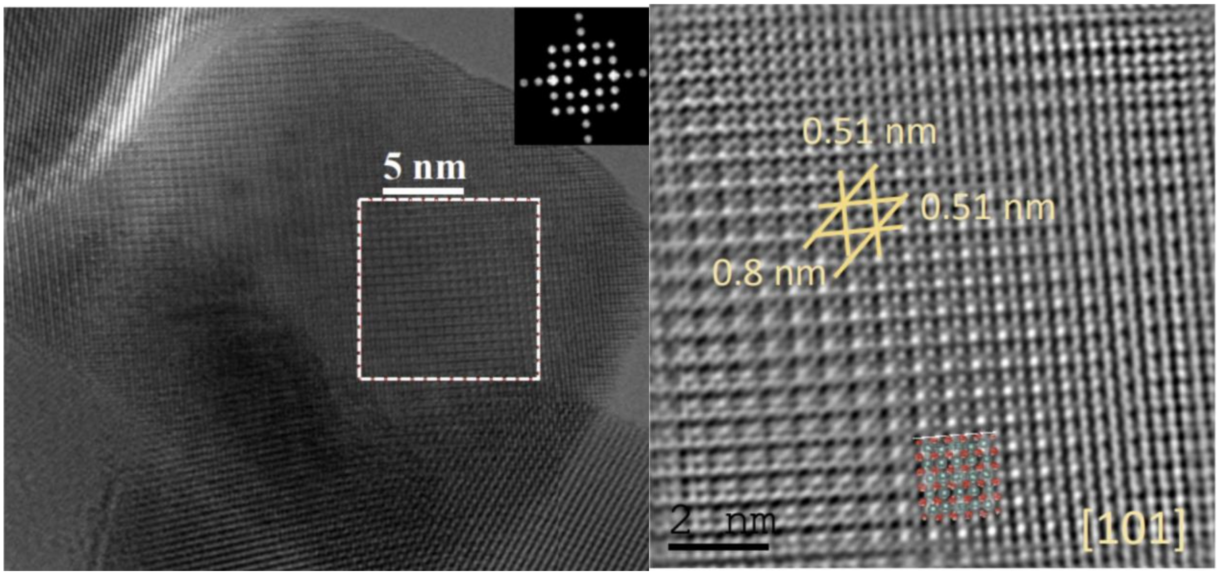

Fig. S4. High resolution TEM images of nanoparticles

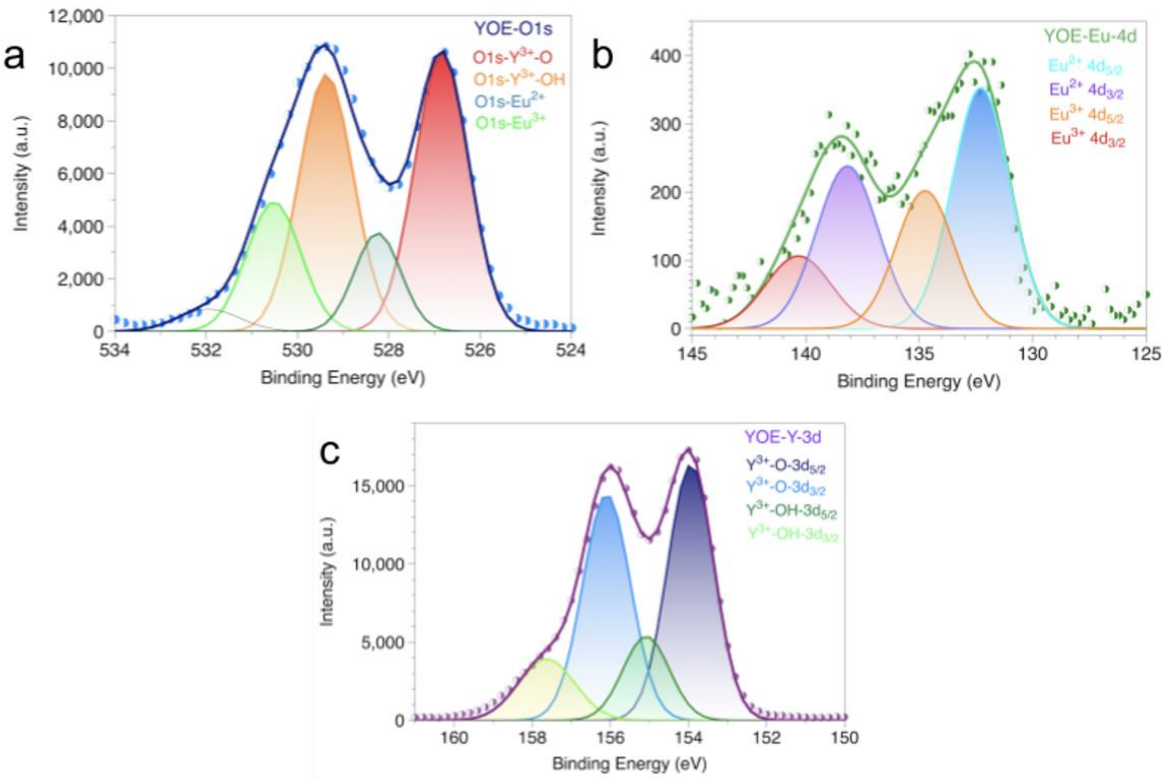

Fig. S5. Fitted high resolution XPS spectra of (a) O1s, (b) Eu-4d, and (c) Y-3d of Y2O3:Eu nanoparticles

	0 h	24 h	72 h
H_2O	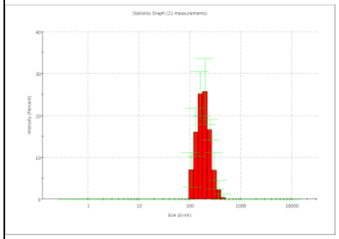 176.3±34.98nm	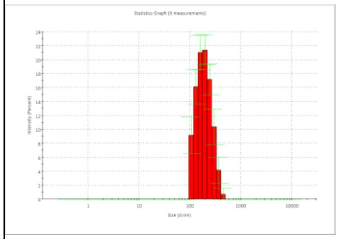 176.7±48.67nm	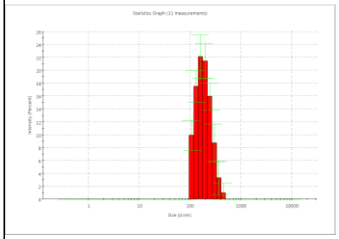 170.9±43.57nm
PBS	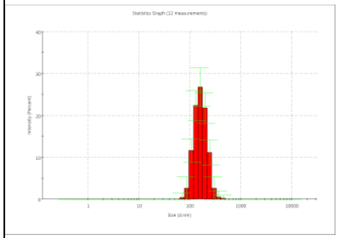 162.4±36.95nm	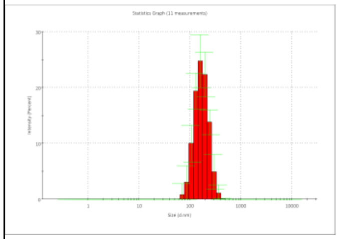 168.8±54.39nm	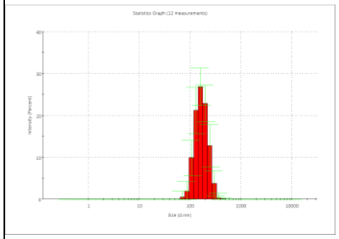 169.2±49.99nm
2% BSA	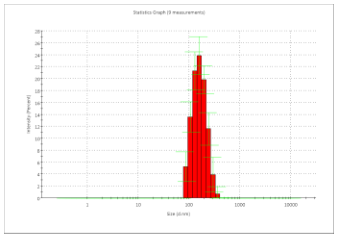 162.5±52.61nm	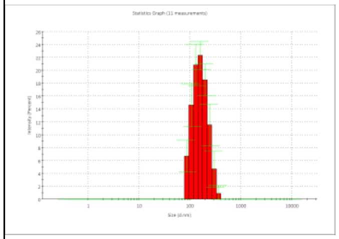 167.2±49.42nm	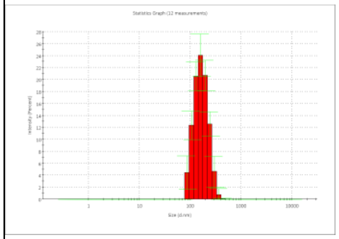 166.1±38.50nm

Fig. S6. DLS stability tracked by observing intensity-based particle size distribution.

	0 h	24 h	72 h
H_2O	-3.15±4.60mV 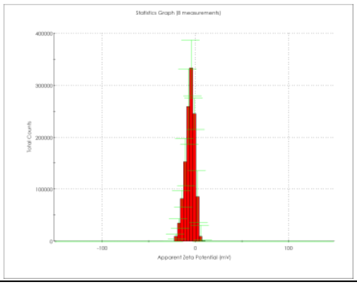	-2.80±4.21mV 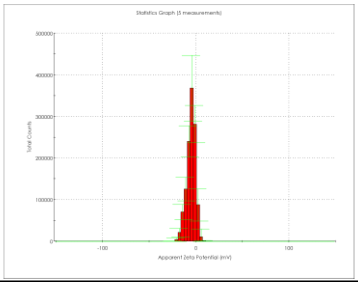	-4.38±6.11mV 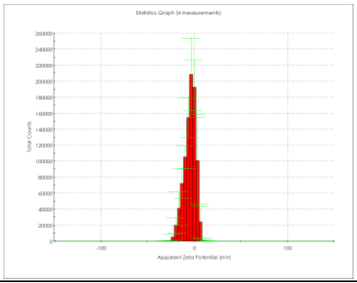
PBS	-3.09±3.78mV 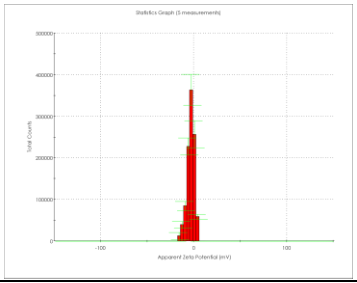	-4.08±6.37mV 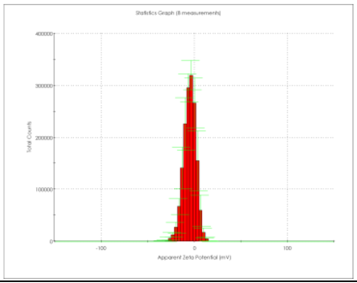	-3.67±5.64mV 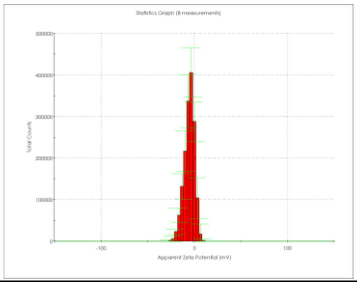
2% BSA	-3.62±5.24mV 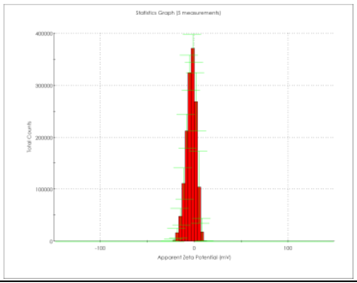	-3.51±5.71mV 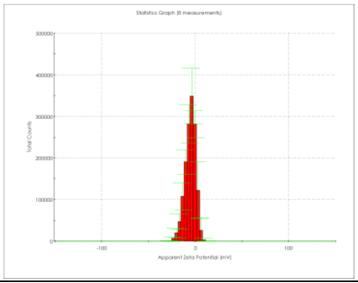	-4.92±5.55mV 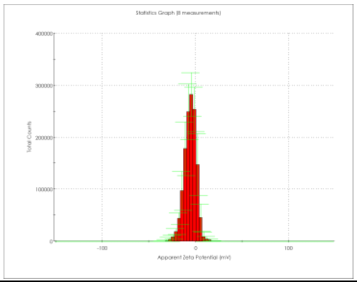

Fig. S7. Stability tracked by observing ζ-potentials**.**

	A long-wave infrared image (wavelengths between 7.2 and 13 microns).	Visible light image	Radiation dose (at 1.12 Gy/min)
	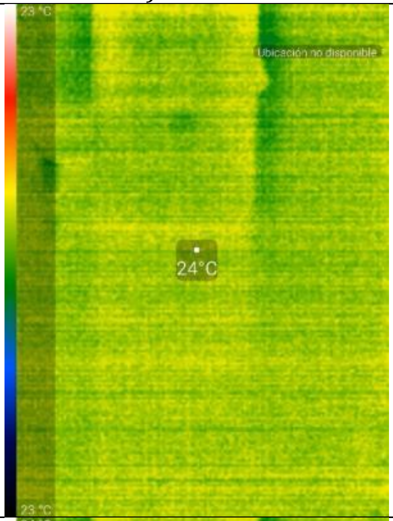	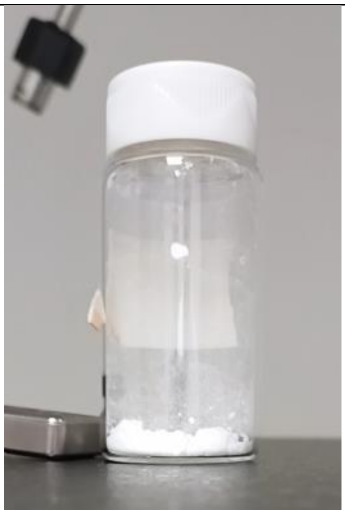	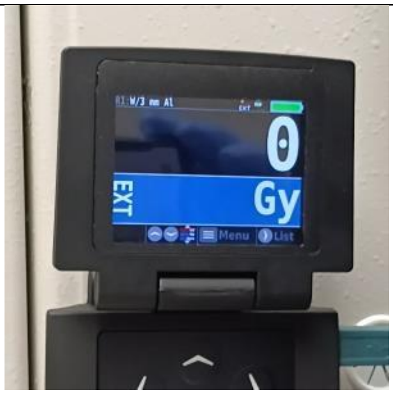
	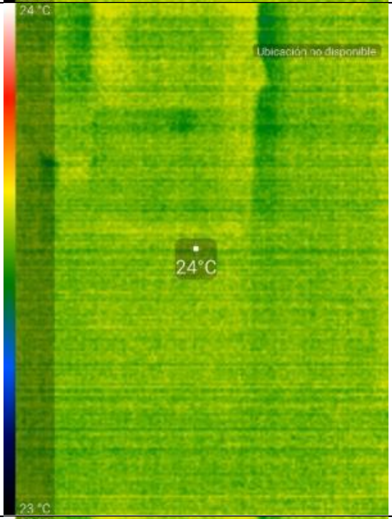	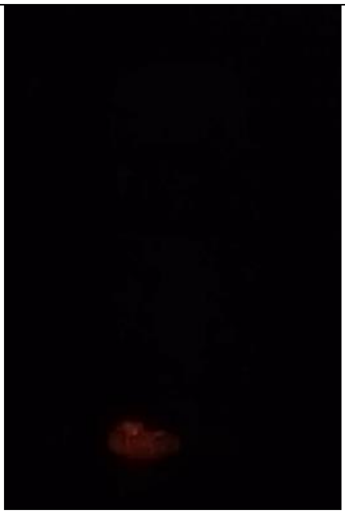	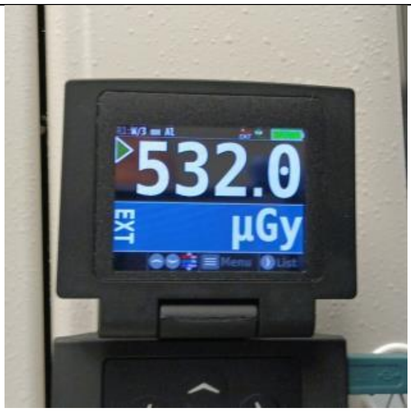
	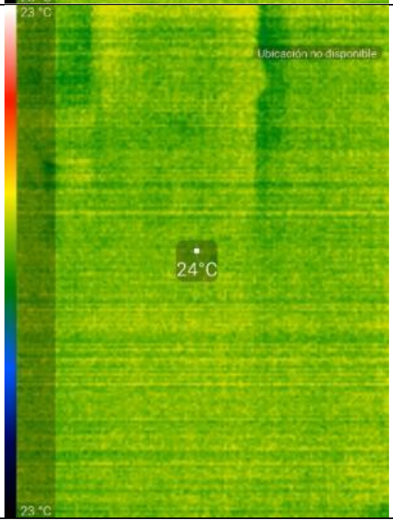	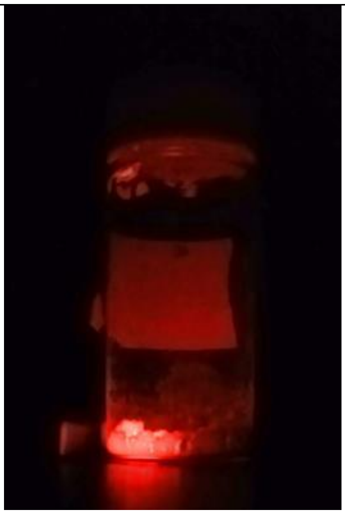	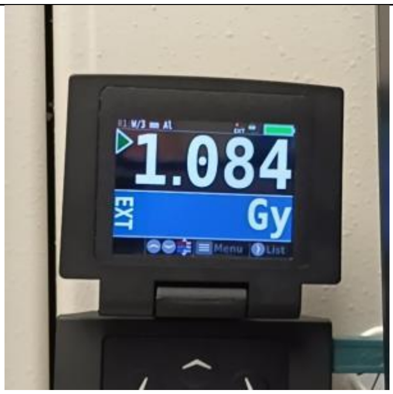

Fig. S8. There was no appreciable increase in temperature with XPDT detected by our infrared camera, pointed directly to the sample (we used a solid sample with the highest light output, and also moved the sample closer to the X-ray source to get the highest intensity).

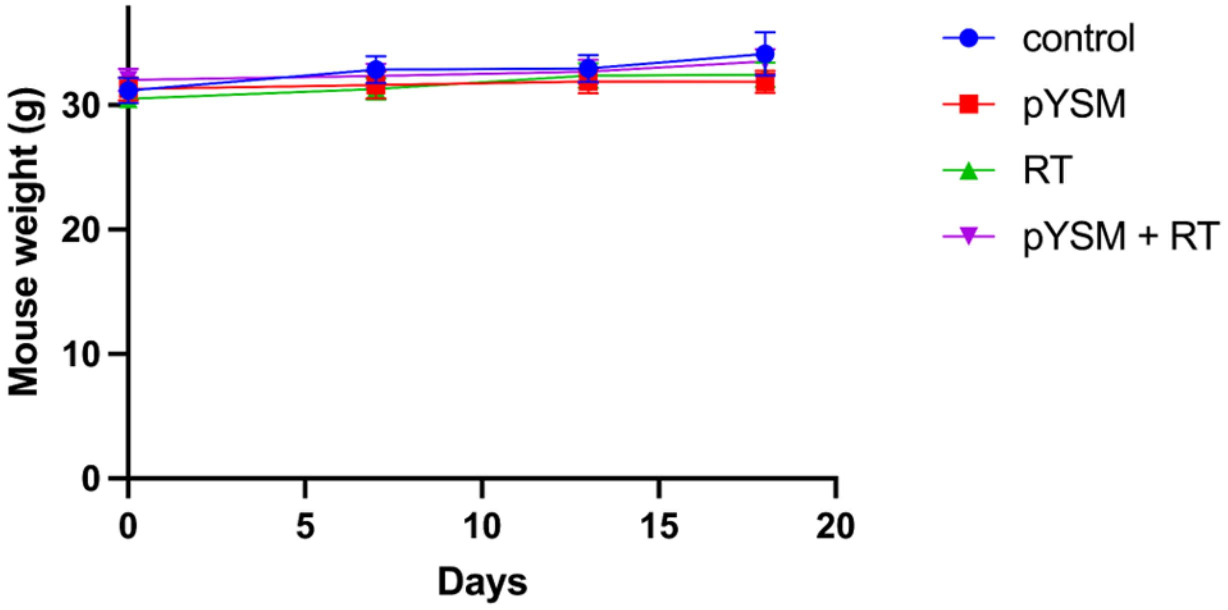

Fig. S9. Mouse weight over time

Table S1. T-cell panel

Antibody	Clone	Fluorophore	Vendor	Catalog No.
CD3	17A2	Brilliant Violet 510	BioLegend	100234
CD4	GK1.5	APC/Cyanine7	BioLegend	100414
CD8	53-6.7	FITC	BioLegend	100706
CD25	PC61	Brilliant Violet 605	BioLegend	102036
Tet+	SIINFEKL	PE	NIH	
IFNg	XMG1.2	Brilliant Violet 421	BioLegend	505830
FOXP3	MF-14	Alexa Fluor 647	BioLegend	126408
Viability		FVS700	BD Biosciences	564997

Table S2. M-cell panel

Antibody	Clone	Fluorophore	Vendor	Catalog No.
CD45	30-F11	Alexa Fluor 700	BioLegend	103128
CD14	REA934	PE-Vio 770	Miltenyi Biotec	130-115-560
CD11b	M1/70	Alexa Fluor 488	Invitrogen	53-0112-82
CD11c	N418	Alexa Fluor 594	BioLegend	117346
F4/80	Cl:A3-1	APC	Bio-Rad	MCA497APC
CD86	GL-1	Brilliant Violet 421	BioLegend	105032
ARG1	A1exF5	PE	Invitrogen	12-3697-82
GR1	RB6-8C5	APC/Fire 750	BioLegend	108456
Viability		Fixable Aqua	BD Biosciences	L34957

Table S3. Gating strategy

Phenotype	Markers
T cells	CD3+
Cytotoxic T cells	CD8+
Activated cytotoxic T cells	CD8+ IFNg+
Antigen-specific T cells	CD8+ Tetramer+ (SIINFEKL)
T-helper cells	CD4+
T-regulatory cells (Tregs)	CD4+ CD25+ FOXP3+
TH1 cells	CD4+IFNg+
M1 macrophages	CD11b+ F4/80+ CD86+ Arg-
M2 macrophages	CD11b+ F4/80+ CD86+-Arg+
Myeloid derived suppressor cells	CD11b+ Gr1+
